# Supplementary material for: Ultrasensitive Fluorescence Lateral Flow Assay for Simultaneous Detection of Pseudomonas aeruginosa and Salmonella typhimurium via Wheat Germ Agglutinin-Functionalized Magnetic Quantum Dot Nanoprobe
Source: Biosensors (Basel). 2022 Oct 31;12(11):942. doi: 10.3390/bios12110942 (PMC9687718; doi:10.3390/bios12110942)
Supplement: Supplementary file 1 [file biosensors-12-00942-s001.zip › biosensors-1969023-supplementary.pdf]

Supporting Information

Ultrasensitive fluorescence lateral flow assay for simultaneous detection of *Pseudomonas aeruginosa* and *Salmonella typhi-murium* via wheat germ agglutinin-functionalized magnetic quantum dot nanoprobe

Zhijie Tu [1,2,†], Xingsheng Yang [1,†], Hao Dong [4], Qing Yu [1,3], Shuai Zheng [3,4], Xiaodan Cheng [1], Chongwen Wang [1,2,3,*], Zhen Rong [1,*] and Shengqi Wang [1,*]

1 Beijing Institute of Microbiology and Epidemiology, Beijing 100089, China
2 Medical Technology School, Xuzhou Medical University, Xuzhou 221004, China
3 College of Life Sciences, Anhui Agricultural University, Hefei 230036, China
4 Science Island Branch of Graduate School, University of Science and Technology of China, Hefei 230036, China
* Correspondence: wangchongwen1987@126.com (C.W.); rongzhen0525@sina.com (Z.R.); sqwang@bmi.ac.cn (S. W.)
† These authors contributed equally to this work.

S1. Experimental section

S1.1 materials and chemicals

Water-soluble CdSe/ZnS-COOH QDs were obtained from Mesolight Inc. (Suzhou, China). Lectin from Triticum vulgaris (wheat germ agglutinin, WGA), glycine, polyethylenimine (PEI) branched (MW 25 kDa), polyvinylpyrrolidone (PVP, 40 K), 2-(N-morpholino) ethanesulfonic (MES), phosphate-buffered saline (PBS), N-(3-dimethyaminopropy)-N′-ethylcarbodiimide hydrochloride (EDC), fetal bovine serum (FBS), N-hydroxysulfosuccinimide sodium salt (sulfo-NHS), and Tween-20 were purchased from Sigma-Aldrich (USA). Goat anti-mouse IgG antibody were purchased from Fitzgerald (America). Mouse monoclonal anti-*P. aeruginosa* antibody was supplied by ThermoFisher (USA) and Mouse monoclonal anti-*S. typhimurium* antibody were purchased from Abcam (UK). Sample pad, absorbent pad, conjugate pad, PVC bottom plate were bought from Jieyi Biotechnology Co. (Shanghai, China) and Nitrocellulose (NC) membrane (CN95) was obtained from Sartorius (Spain). All the bacteria used in this study were supplied by Pro. Shengqi Wang’s group of Beijing Key Laboratory of New Molecular Diagnosis Technologies for Infectious Diseases.

S1.2 Instruments

Transmission electron microscopy (TEM) images were taken on Hitachi H-7650 TEM. Scanning electron microscopy (SEM) images were obtained by JEOL JSM-7001F microscope (JEOL, Japan). Elemental mapping images were recorded by energy-dispersive X-ray spectroscopy (EDS) using a Philips Tecnai G2 F20 microscope equipped with a STEM unit. Zeta potential was measured with Mastersizer 2000 (Malvern, UK). Fourier transform infrared (FTIR) spectroscopies were measured via FTIR spectroscopy (Thermo Fisher Scientific, Inc., Waltham, MA). The fluorescence signals of test lines were simultaneously read by using a portable FIC-S1 fluorescence reader (365 nm excitation/610 nm emission), which were purchased from Suzhou Hemai Precision Instrument Co., Ltd (China).

S1.3. Preparation of immuno-Mag@QD-LFA

The conjugation of Mag@QD and anti-*P. aeruginosa* antibodies was conducted via the carbodiimide chemistry. First, 1mL of Mag@QD (1 mg/mL) was resuspended in 500 μL of MES buffer (100 mM, pH 5.5) with an external magnetic field, and then mixed with 5 μL of EDC (0.1 M) and 10 μL of sulfo-NHS (0.1 M). After sonicating for 15 min, the mixture was washed to remove the excess activators (EDC/sulfo-NHS) and dispersed in 200 μL of PBS buffer (10 mM, pH 7.4). Then, the activated Mag@QD solution was incubated with 20 μg of *P. aeruginosa* antibodies for 2 h, followed by surface blocking with 100 μL of 10% BSA solution for 1 h. The antibody-conjugated Mag@QD were magnetically collected and resuspended with 200 μL of PBS solution (containing 0.05% Tween-20)

S1.4. Detection of bacteria by using immuno-Mag@QD-LFA

The detection assay for *P. aeruginosa* was performed in a 1.5 mL EP tube and on a test strip. First, 5 μL of immune-Mag@QD were added into 1 mL of sample solution, and the mixture was incubated for 15 min at room temperature. Then, the mixed immune- Mag@QD-bacteria complexes were rapidly separated by using an

external magnet and resuspended in 100 μL of running buffer (10 mM PBS, pH 7.4, 10% FBS, 0.5%milk, 1% Tween 20). Finally, the running buffer mixture were loaded onto the sample pad of tested strip to start the chromatographic reaction. After 15 min, the fluorescence intensity on the test line was measured by a portable fluorescence reader.

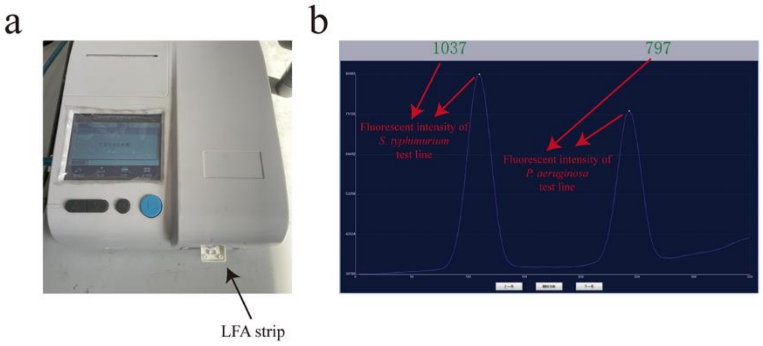

Figure S1 (a) Photograph of fluorescence reader. (b) The fluorescence intensity read by the fluorescence reader.

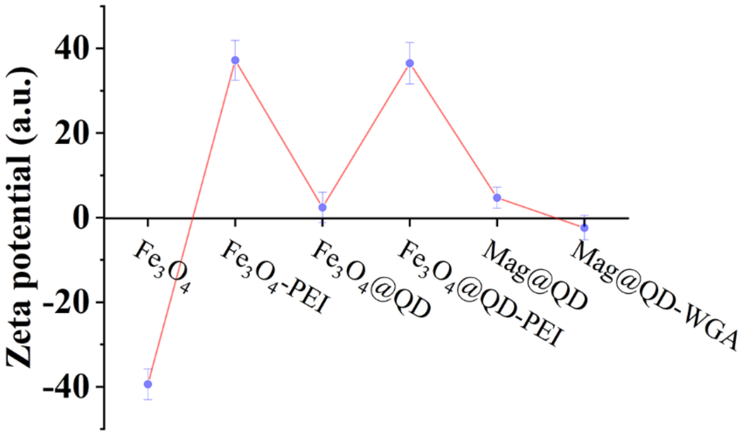

Figure S2 Zeta potentials of the products from each step.

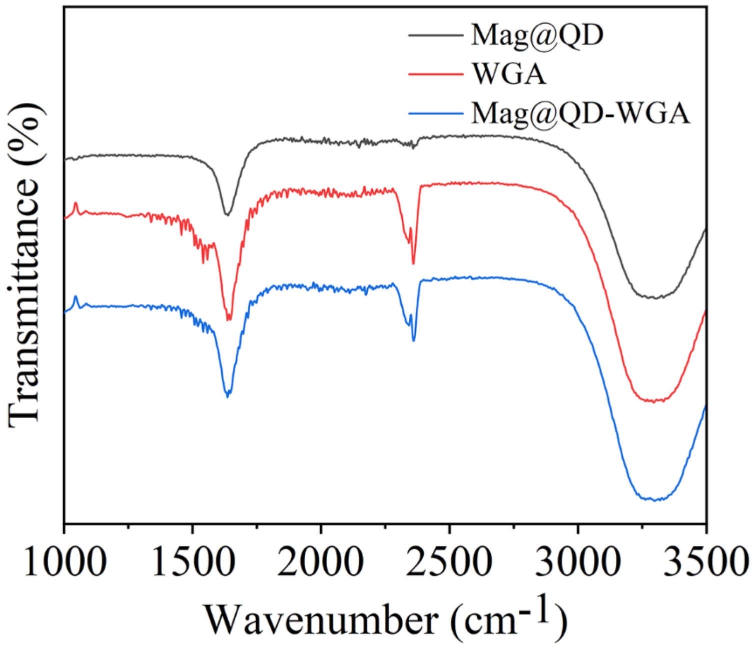

Figure S3 FTIR spectra of Mag@QD (black line), WGA (red line), and Mag@QD-WGA (blue line). The characteristic absorption peaks corresponding to protein amide bands I (2339 cm^{-1}) and II (2354 cm^{-1}) appearing in Mag@QD-WGA spectra reveal the successful coupling of WGA.

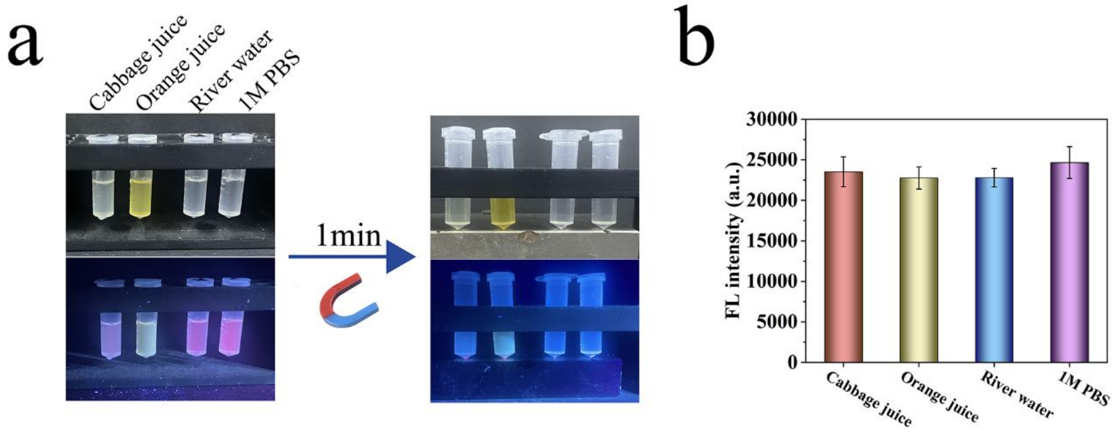

Figure S4 (a) Magnetic separation behavior and (b) fluorescence intensity of Mag@QD-WGA in different samples (cabbage juice, orange juice, river water, and PBS). The error bars are calculated from three experiments.

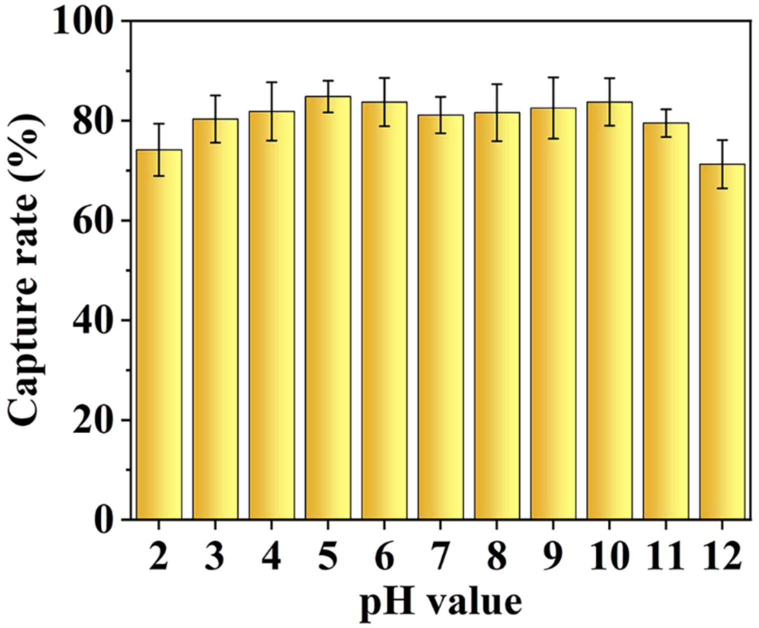

Figure S5 Capture abilities of Mag@QD-WGA for *P. aeruginosa* in aqueous solution with different pH values. The error bars are calculated from three experiments.

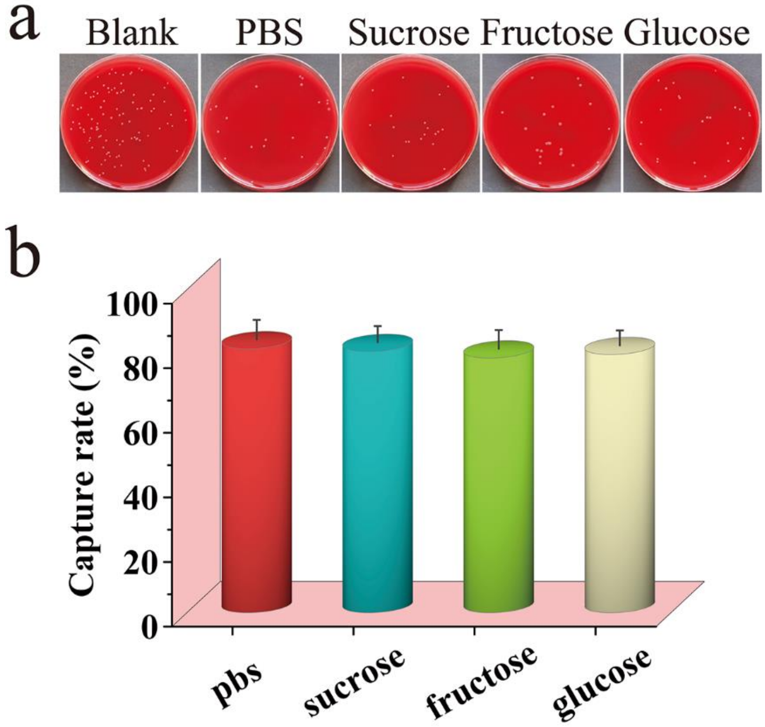

Figure S6 (a) Photographs of the colonies on blood agar plates indicating the amounts of remaining *P. aeruginosa* in the supernatant after the magnetic enrichment of Mag@QD-WGA in PBS and three different sugar solutions. (b)

Corresponding capture ability of Mag@QD-WGA in PBS and three different sugar solutions. The error bars are calculated from three experiments.

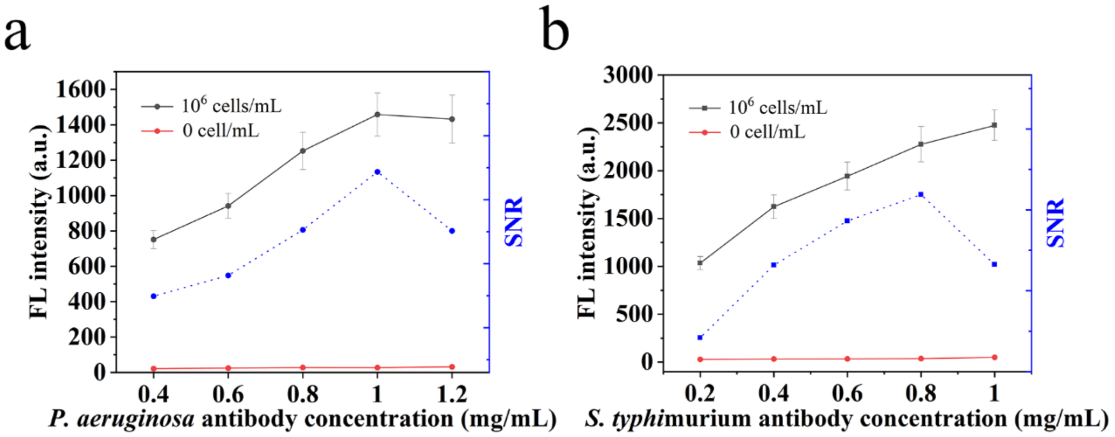

Figure S7 Optimization of capture antibody concentration on test line for (a)*P. aeruginosa* and (b) *S. typhimurium*. The error bars are calculated from three experiments.

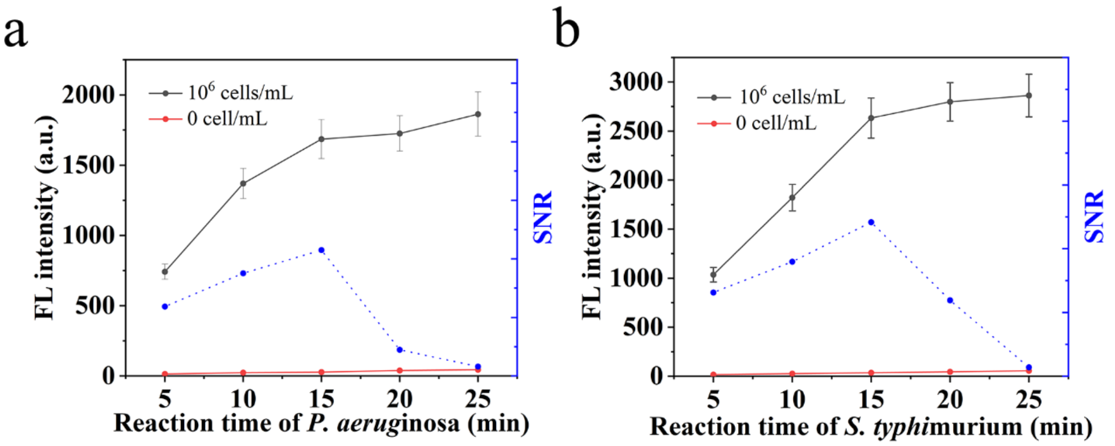

Figure S8 Optimization of (a) *P. aeruginosa* and (b) *S. typhimurium* reaction time on the test line for the LFA detection system. The error bars are calculated from three experiments.

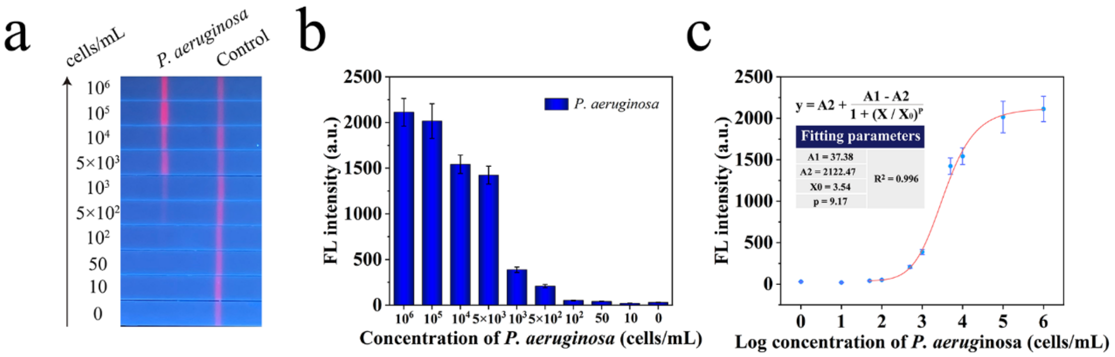

Figure S9 (a) Photograph of LFA strip with C line for *P. aeruginosa*. (b) Corresponding fluorescence intensity of test lines for different concentrations of *P.*

aeruginosa. (c) Corresponding calibration line of *P. aeruginosa*. The error bars are calculated from three experiments.

Table S1. Recovery results of the bacteria in river water, cabbage juice, and orange juice.

Sample	Spiked/Detected (cells/mL)		Recovery (%)		RSD (%)	
	P. aeruginosa	*S. typhimurium*	*P. aeruginosa*	*S. typhimurium*	*P. aeruginosa*	*S. typhimurium*
River water	$10^6/1.07\times10^6$	$10^6/0.99\times10^6$	107.35	98.91	7.99	4.82
	$10^4/0.94\times10^4$	$10^4/0.95\times10^4$	93.7	95.48	6.51	8.13
	$10^2/1.06\times10^2$	$10^2/1.01\times10^2$	105.8	101.03	7.05	8.52
Cabbage juice	$10^6/0.94\times10^6$	$10^6/0.92\times10^6$	93.91	91.64	8.12	6.6
	$10^4/0.97\times10^4$	$10^4/0.90\times10^4$	97.49	90.19	9.23	10.39
	$10^2/1.00\times10^2$	$10^2/0.99\times10^2$	99.66	99.19	7.3	9.98
Orange juice	$10^6/0.97\times10^6$	$10^6/0.95\times10^6$	96.57	94.71	6.29	6.23
	$10^4/1.05\times10^4$	$10^4/1.10\times10^4$	104.96	109.51	9.51	6.57
	$10^2/1.03\times10^2$	$10^2/0.96\times10^2$	102.75	95.64	9.78	8.92

Table S2 Comparison of Mag@QD-WGA-LFA with other reported multi LFA.

Detection method	Bacteria	Recognition molecules	LODs (cells/mL)	Reference
Fluorescent-LFA	*Salmonella* spp./*E. coli* O157:H7	Dual antibodies sandwich complex	$10^5/10^5$	[1]
Colorimetric-LFA	*S. boydii*/*E. coli* O157:H7	Dual antibodies sandwich complex	$10^6/10^6$	[2]
Colorimetric-LFA	*Klebsiella*/*Raoultella*	Dual antibodies sandwich complex	$10^4/10^5$	[3]
Fluorescent-LFA	*E. coli* O157:H7/*S. typhimurium*	Dual antibodies sandwich complex	50/50	[4]
Fluorescent-LFA	*S. pneumoniae*/*S. aureus*	Dual antibodies sandwich complex	13/20	[5]
Fluorescent-LFA	*P. aeruginosa*/*S. typhimurium*	WGA and antibody	25/28	This work

References

1. Rajendran, V. K.; Bakthavathsalam, P.; Jaffar Ali, B. M., Smartphone based bacterial detection using biofunctionalized fluorescent nanoparticles. Microchimica Acta 2014, 181, 1815–1821.

2. Song, C.; Liu, C.; Wu, S.; Li, H.; Guo, H.; Yang, B.; Qiu, S.; Li, J.; Liu, L.; Zeng, H.; Zhai, X.; Liu, Q., Development of a lateral flow colloidal gold immunoassay strip for the simultaneous detection of Shigella boydii and Escherichia coli O157:H7 in bread, milk and jelly samples. *Food Control* **2016,** *59*, 345–351.

3.Tominaga, T., Rapid detection of Klebsiella pneumoniae, Klebsiella oxytoca, Raoultella ornithinolytica and other related bacteria in food by lateral-flow test strip immunoassays. *J Microbiol. Methods* **2018,** *147*, 43–49.

4. Zheng, S.; Yang, X.; Zhang, B.; Cheng, S.; Han, H.; Jin, Q.; Wang, C.; Xiao, R., Sensitive detection of Escherichia coli O157:H7 and Salmonella typhimurium in food samples using two-channel fluorescence lateral flow assay with liquid Si@quantum dot. *Food Chem* **2021,** *363*, 130400.

5. Cheng, X.; Zheng, S.; Wang, W.; Han, H.; Yang, X.; Shen, W.; Wang, C.; Wang, S., Synthesis of two-dimensional graphene oxide-fluorescent nanoprobe for ultrasensitive and multiplex immunochromatographic detection of respiratory bacteria. *Chemical Engineering Journal* **2021,** *426*.
